# Ossification of the Choroid; Eneucleation of the Globe

**Published:** 1871-03

**Authors:** C. Hixson

**Affiliations:** Formerly Professor of Ophthalmology in the College of Physicians and Surgeons, Kansas City, Mo.


### OSSIFICATION OF THE CHOROID; ENEUCLEATION OF THE GLOBE.

By C. HIXSON, M.D., formerly Professor of Ophthalmology in the College of Physicians and Surgeons, Kansas City, Mo.

More than twenty years since, a Mr. T. J. Ford, who at that time resided in the State of Kentucky, whilst exploding a percussion cap with a hammer, received a piece of it in his left eye. Severe inflammation followed, and, after having suffered untold misery, he was induced to seek surgical aid. Amongst others, Prof. Gross, then of Louisville, was consulted, who advised the removal of the eye. To this the patient would not accede. He finally removed to this State, and the continued pain, and failing vision of the other eye, induced him to submit to the removal of the injured organ.

Accordingly, on the 25th of January, I eneucleated the globe. Examination of the eye after removal revealed the interesting condition of ossification of the choroid, embracing fully three-fourths of the entire area of the first division of that membrane, or that part extending from the optic nerve entrance to the ciliary processes. Nothing but a mere trace of the choroidal pigment was found remaining over the ossified portion. Under the magnifiers the ossific structure presents a most beautiful specimen of bone, presenting perfect lacunæ, and occasional large openings which, perhaps, transmitted the choroidal vessels. A piece of the copper-cap, of the size of half a pin's head, was found imbedded in what was, originally, the lens, but which had become so mixed up with, and so adherent to, the posterior surface of the cornea as to be inseparable from it. The aqueous chamber was, of course, entirely obliterated, and the cornea opake. The wasted retina was found in threads, the optic papilla atrophied, and the optic nerve soft and shrunken.

Ossification of the choroid seems to be an infrequent pathological condition. I so infer from the scarcity of cases reported through the current literature of the eye, as well as the small space alloted to its consideration in works specially devoted to this department of medicine. It has happened in my experience to meet but one case of this kind.

That this was a case of true ossification *within* the stroma of the choroid there can, I think, be no doubt, as nothing was left but a mere trace of the pigment from the optic nerve entrance to the ciliary processes.

